# Potentiation of the anti-tumour effects of Photofrin®-based photodynamic therapy by localized treatment with G-CSF

**DOI:** 10.1054/bjoc.1999.1078

**Published:** 2000-03-21

**Authors:** J Golab, G Wilczynski, R Zagozdzon, T Stoklosa, A Dabrowska, J Rybczynska, M Wasik, E Machaj, T Oldak, K Kozar, R Kaminski, A Giermasz, A Czajka, W Lasek, W Feleszko, M Jakobisiak

**Affiliations:** Department of Immunology, Institute of Biostructure, Chałubińskiego 5, Warsaw, 02-004, Poland; , Department of Pathology, Chałubińskiego 5, Warsaw, 02-004, Poland; Transplantation Institute, Medical University of Warsaw, Nowogrodzka 59, Warsaw, 02-006, Poland; Department of Laboratory Diagnostics and Clinical Immunology, Medical University of Warsaw, Marszałkowska 24, Warsaw, 00-576, Poland; Department of Experimental Hematology, Maria Skłodowska-Curie Memorial Cancer Center, Institute of Oncology, Roentgena 5, Warsaw, 02-781, Poland; Department of Radiation Hematology, WIHiE, Szaserów 128, Warsaw, 04-363, Poland

**Keywords:** photodynamic therapy, Photofrin^®^, G-CSF, neutrophils

## Abstract

Photofrin^®^-based photodynamic therapy (PDT) has recently been approved for palliative and curative purposes in cancer patients. It has been demonstrated that neutrophils are indispensable for its anti-tumour effectiveness. We decided to evaluate the extent of the anti-tumour effectiveness of PDT combined with administration of granulocyte colony-stimulating factor (G-CSF) as well as the influence of Photofrin^®^and G-CSF on the myelopoiesis and functional activity of neutrophils in mice. An intensive treatment with G-CSF significantly potentiated anti-tumour effectiveness of Photofrin^®^-based PDT resulting in a reduction of tumour growth and prolongation of the survival time of mice bearing two different tumours: colon-26 and Lewis lung carcinoma. Moreover, 33% of C-26-bearing mice were completely cured of their tumours after combined therapy and developed a specific and long-lasting immunity. The tumours treated with both agents contained more infiltrating neutrophils and apoptotic cells then tumours treated with either G-CSF or PDT only. Importantly, simultaneous administration of Photofrin^®^and G-CSF stimulated bone marrow and spleen myelopoiesis that resulted in an increased number of neutrophils demonstrating functional characteristics of activation. Potentiated anti-tumour effects of Photofrin^®^-based PDT combined with G-CSF observed in two murine tumour models suggest that clinical trials using this tumour therapy protocol would be worth pursuing. © 2000 Cancer Research Campaign


*British Journal of Cancer* (2000) **82**(8), 1485–1491
© 2000 Cancer Research Campaign
DOI: 10.1054/ bjoc.1999.1078, available online at http://www.idealibrary.com on IDEAL®

### Potentiation of the anti-tumour effects of Photofrin®-based photodynamic therapy by localized treatment with G-CSF

**J Gołąb[1], G Wilczyński[2], R Zagożdżon[1,3], T Stokłosa[1], A Dąbrowska[1], J Rybczyńska[4], M Wąsik[4], E Machaj[5], T Ołdak[6], K Kozar[1], R Kamiński[1], A Giermasz[1], A Czajka[1], W Lasek[1], W Feleszko[1] and M Jakóbisiak[1]**

[1]Department of Immunology, Institute of Biostructure, Chałubińskiego 5, 02-004 Warsaw, Poland; [2]Department of Pathology, Chałubińskiego 5, 02-004 Warsaw, Poland; [3]Transplantation Institute, Medical University of Warsaw, Nowogrodzka 59, 02-006 Warsaw, Poland; [4]Department of Laboratory Diagnostics and Clinical Immunology, Medical University of Warsaw, Marszałkowska 24, 00-576 Warsaw, Poland; [5]Department of Experimental Hematology, Maria Skłodowska-Curie Memorial Cancer Center, Institute of Oncology, Roentgena 5, 02-781 Warsaw, Poland; [6]Department of Radiation Hematology, WIHiE, Szaserów 128, 04-363 Warsaw, Poland

**Summary** Photofrin®-based photodynamic therapy (PDT) has recently been approved for palliative and curative purposes in cancer patients. It has been demonstrated that neutrophils are indispensable for its anti-tumour effectiveness. We decided to evaluate the extent of the anti-tumour effectiveness of PDT combined with administration of granulocyte colony-stimulating factor (G-CSF) as well as the influence of Photofrin® and G-CSF on the myelopoiesis and functional activity of neutrophils in mice. An intensive treatment with G-CSF significantly potentiated anti-tumour effectiveness of Photofrin®-based PDT resulting in a reduction of tumour growth and prolongation of the survival time of mice bearing two different tumours: colon-26 and Lewis lung carcinoma. Moreover, 33% of C-26-bearing mice were completely cured of their tumours after combined therapy and developed a specific and long-lasting immunity. The tumours treated with both agents contained more infiltrating neutrophils and apoptotic cells then tumours treated with either G-CSF or PDT only. Importantly, simultaneous administration of Photofrin® and G-CSF stimulated bone marrow and spleen myelopoiesis that resulted in an increased number of neutrophils demonstrating functional characteristics of activation. Potentiated anti-tumour effects of Photofrin®-based PDT combined with G-CSF observed in two murine tumour models suggest that clinical trials using this tumour therapy protocol would be worth pursuing. © 2000 Cancer Research Campaign

**Keywords**: photodynamic therapy; Photofrin®; G-CSF; neutrophils

Photodynamic therapy (PDT) with Photofrin® is a promising treatment modality for the management of a variety of solid neoplasms. This modality was recently approved for the treatment of oesophageal, lung, bladder, gastric and cervical cancers in various countries (Dougherty et al, 1998). Clinical trials using PDT for the treatment of cancers of other sites, including gastrointestinal tract, are also being conducted (Dougherty et al, 1998; Kashtan et al, 1996). Although most of the regimens use PDT for palliative treatment, it seems increasingly evident that this therapy might also be useful in the treatment of a wide range of cancers, from early to late stages (Reynolds, 1997).

PDT with Photofrin® involves the systemic administration of a photosensitizer followed, after a time necessary for accumulation of the sensitizer in the tumour, by the illumination of the tumour area with visible light in the red or near-infrared spectral region (Pass, 1993). There is an ample evidence that PDT, besides eliciting direct cytotoxic effects towards tumour cells, involving formation of reactive oxygen species (Henderson and Dougherty, 1992), also induces indirect anti-tumour effects resulting from deprivation of oxygen and nutrients due to vaso-occlusion (Ochsner, 1997). PDT is also capable of inducing apoptosis in tumour cells (Granville et al, 1997; Ochsner, 1997; Ahmad et al, 1998). Moreover, PDT elicits non-specific and specific immune responses against the tumour (Korbelik and Dougherty, 1999). Endothelial cells seem to play a central role in these immune responses. Besides constriction at the time of PDT exposure (Fingar et al, 1992), endothelial cells facilitate platelet aggregation and thrombus formation that eventually lead to blood flow stasis (Ben-Hur et al, 1988; Foster et al, 1991; Fingar et al, 1997). Simultaneously, intensive extravasation of leucocytes, especially neutrophils and macrophages, into the tumour tissue is observed (Krosl et al, 1995; Gollnick et al, 1997). These cells accumulate in the tumour area as early as 5 min after PDT (Fingar et al, 1992), where they may kill tumour cells directly (Faddy et al, 1990) or indirectly through cooperation with lymphoid cells (Stoppacciaro et al, 1993; Zilocchi et al, 1998).

Recent studies have demonstrated that the anti-tumour efficacy of PDT can be improved by the administration of macrophage activating factors such as granulocyte-macrophage colony-stimulating factor (GM-SCF) (Krosl et al, 1996) or modified vitamin $D_3$-binding protein (Korbelik et al, 1997). Moreover, PDT efficacy is attenuated in immune-deficient nude and severe combined immune deficient (SCID) mice, implying the immune mechanisms among the effectors of PDT (Korbelik et al, 1996). It has also been observed that neutrophils are indispensable for successful PDT

*Received 22 July 1999*
*Revised 11 November 1999*
*Accepted 17 November 1999*

*Correspondence to:* J Gołąb

(de Vree et al, 1996*a*). Specifically, depletion of neutrophils in tumour-bearing mice using polyclonal anti-granulocytes antibodies (de Vree et al, 1996*a*), or blocking functions of cell adhesion molecules engaged in the recruitment of these leucocytes in tissues by anti-CD18 monoclonal antibody (de Vree et al, 1996*b*), was found to decrease PDT-mediated anti-tumour effects. These observations prompted us to investigate the extent to which intensive G-CSF therapy, which results in increased production of neutrophils, is capable of potentiating the anti-tumour efficacy of PDT in mice.

### MATERIALS AND METHODS

### Mice

(C57BL/6 × DBA/2)$F_1$ mice, hereafter called B6D2$F_1$, and Balb/c mice, 8–12 weeks of age, were used in the experiments. Breeding pairs were obtained from the Inbred Mice Breeding Center of the Institute of Immunology and Experimental Medicine (Wrocław, Poland), and from the Institute of Oncology, (Warsaw, Poland). All experiments with animals were performed in accordance with the guidelines approved by the Ethical Committee of the Medical University of Warsaw.

### Reagents

Photofrin®, was generous gift of QLT PhotoTherapeutics, Inc. (Vancouver, BC, Canada). Recombinant human G-CSF (Neupogen) was purchased from Hoffman-La Roche (Basel, Switzerland). G-CSF was diluted with 0.1% bovine serum albumin (BSA; Sigma, St. Louis, MO, USA) in phosphate-buffered saline (PBS) for in vivo experiments.

### Tumours

Colon-26 (C-26), a poorly differentiated and immunogenic colon adenocarcinoma cells, and a non-immunogenic Lewis lung (3LL) carcinoma cells were used. Cells were cultured in RPMI-1640 medium (Gibco-BRL, Paisley, UK) supplemented with 10% heat-inactivated fetal calf serum (FCS), antibiotics, 2-mercaptoethanol (50 μM) and L-glutamine (2 mM) (all from Gibco-BRL), hereafter referred to as culture medium. For in vivo experiments exponentially growing tumour cells were harvested, resuspended in PBS medium to appropriate concentration of cells and injected ($1 \times 10^5$ C-26 cells or $5 \times 10^5$ 3LL cells in 20 μl PBS) into the footpad of the right hind limb of experimental mice. Tumour cell viability ranged between 96 and 97%.

### Tumour treatment and monitoring

Tumour-bearing mice were treated with G-CSF intratumourally (i.t.) starting from day 5 following inoculation of C-26 or 3LL cells. G-CSF was given twice daily, every 12 h, for 5 consecutive days, at a dose of 1 μg adjusted to 20 μl in 0.1% BSA-PBS. Mice in control groups were injected with 20 μl 0.1% BSA-PBS i.t. in the same regimen as the treated mice.

Photofrin® was administered intraperitoneally (i.p.) at a dose of 15 mg $kg^{-1}$ 24 h before illumination with 630-nm light (day 4 of G-CSF administration, day 9 of the experiment). The light source was a He-Ne ion laser (Amber, Warsaw, Poland). The light was delivered on day 10 of the experiment using a fibreoptic light delivery system. The power density at the illumination area, which encompassed the tumour and 1–1.5 mm of the surrounding skin, was approximately 80 mW $cm^{-2}$ (40 mW laser output). The total light dose delivered to the tumours was 150 J $cm^{-2}$. During the light treatment mice were anaesthetized with chloral hydrate (3.6 mg $kg^{-1}$) and restrained in a specially designed holder. Local tumour growth was determined as described (Golab et al, 1998*a*) by the formula:

Tumour volume ($mm^3$) =
(longer diameter) × (shorter diameter)$^2$

Relative tumour volume was calculated as follows:

Relative tumour volume =
[(tumor volume) ÷ (initial tumor volume)] × 100%.

Transient tumour oedema that developed after PDT was included in the total volume of measured tumours.

### Histopathology, immunohistochemistry and TUNEL staining

The individual C-26 tumours were excised and snap-frozen 24 h after PDT. Several cryostat sections, 10 μm thick, were made from each tumour. Some sections were stained with haematoxylin and eosin (H&E) routinely, the other sections underwent immunohistochemical procedures and TUNEL staining. Two-step immunohistochemistry was performed, with a primary rat anti-mouse neutrophil monoclonal antibody (clone RB6-8C5, Pharmingen, San Diego, CA, USA) against Gr-1 epitope, and the secondary being fluorescein isothiocyanate (FITC)-labelled goat F(ab´)$_2$ anti-rat IgG (H+L) (Southern Biotechnology Associates, Inc., Birmingham, AL, USA).

DNA fragmentation was detected by means of terminal deoxynucleotide transferase based, in situ cell death detection kit (TUNEL, Boehringer Mannheim, Germany). The procedure was performed according to the manufacturer’s instructions. Deoxynucleotide incorporated in DNA strand breaks was flourescein-labelled.

### Analysis of peripheral blood cells

In the experiments in which the influence of Photofrin® and/or G-CSF on haematopoiesis was studied, Balb/c mice were treated in the same treatment regimen as in the above experiments. G-CSF was given twice daily, every 12 h, for 5 consecutive days, at a dose of 1 μg. Photofrin® was administered at a dose of 15 mg $kg^{-1}$. Mice in control groups were injected with 0.1% BSA-PBS and 5% dextrose. Blood was collected from retroorbital sinus of anaesthetized mice 12 h after the last G-CSF dose and peripheral blood cells were assessed using a Sysmex-820 cell counter (Sysmex, Kyoto, Japan) adapted for the analysis of rodent cells.

### In vitro colony assays

Spleen and bone marrow cells were collected 12 h after the last G-CSF dose and the colony-forming units granulocyte–macrophage (CFU-GM) and the colony-forming units granulocyte, erythrocyte, monocyte, megakaryocyte (CFU-GEMM) assays were performed as described previously (Golab et al, 1998*a*).

 © 2000 Cancer Research Campaign

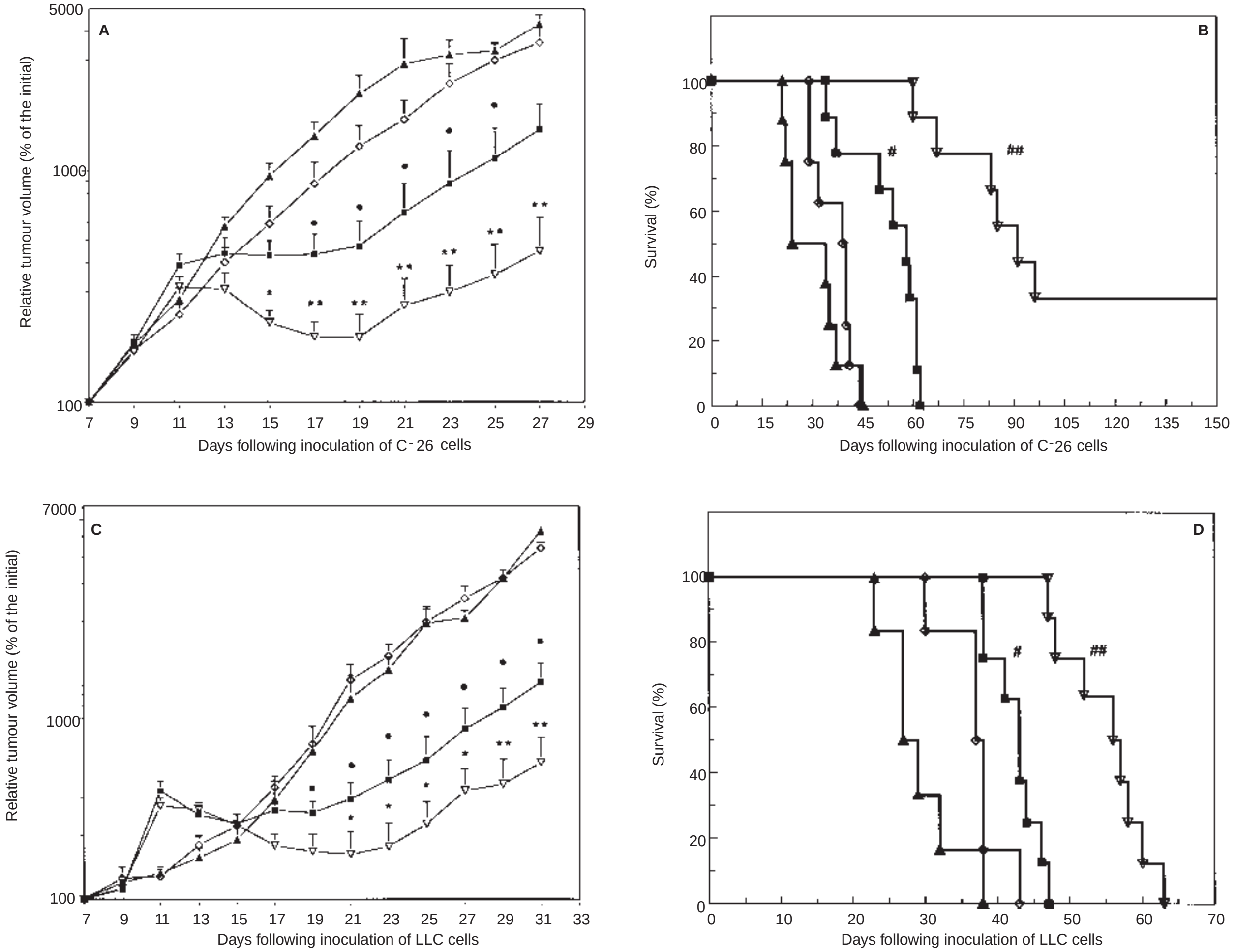

**Figure 1** Anti-tumour effects of the combined treatment with Photofrin®-based PDT and G-CSF. G-CSF was administered i.t. at a dose of 1 µg, every 12 h, for 5 consecutive days, starting from day 5 after inoculation of tumour cells. Photofrin® was administered i.p. at a dose of 15 mg $kg^{-1}$, 24 h before laser illumination (150 J $cm^{-2}$ on day 10 after inoculation of tumour cells). Measurements of tumour diameter started on day 7 after inoculation of tumour cells. (**A**) The influence of the combined treatment on the growth of C-26 tumours in Balb/c mice ($n$ = 8–9). (**B**) Kaplan–Meier plot of the survival of Balb/c mice bearing C-26 tumours. (**C**) The influence of the combined treatment on the growth of 3LL tumours in $B6D2F_1$ mice ($n$ = 6–8). (**D**) Kaplan–Meier plot of the survival of $B6D2F_1$ mice bearing 3LL tumours. •$P < 0.05$ (Mann–Whitney $U$-test) in comparison with control and G-CSF-treated mice. *$P < 0.05$; **$P < 0.01$ (Mann–Whitney $U$-test) in comparison with each of the remaining groups. #$P < 0.05$ (log-rank survival analysis) in comparison with controls and G-CSF treated mice); ##$P < 0.01$ (log-rank survival analysis) in comparison with each of the remaining groups. ▲ – control; ◇ – G-CSF; ■ – PDT; ∇ – PDT + G-CSF

### Luminol-enhanced chemiluminescence measurements

To evaluate the respiratory burst of oxygen-derived metabolites in Balb/c mice treated with G-CSF and/or Photofrin®, the luminol-enhanced chemiluminescence (ECL) was measured in whole peripheral blood collected from retroorbital sinus 12 h after the last G-CSF dose. Mice were treated with G-CSF and/or Photofrin® as described above. Ten microlitres of peripheral blood, five times diluted with Hanks balanced salt solution, was mixed with 200 µl of luminol ($1 \times 10^{-5}$ M; Sigma). The vials were placed in the measuring chamber of a liquid scintillator counter LKB Wallac 1409 with the chemiluminescence option (Pharmacia LKB, Turku, Finland) for the measurement of spontaneous luminol-ECL. Then 20 µl of serum-opsonized zymosan (2 mg $ml^{-1}$; Sigma) was added to vials and the luminol-ECL of zymosan-activated cells was measured using the same apparatus. The results were expressed as counts per minute (cpm).

### Statistical analysis

Data are presented as means ± standard error (s.e.). Differences in tumour volume, chemiluminescence, numbers of bone marrow and spleen colonies and peripheral blood leucocytes were analysed for significance by Student's $t$ test. Additionally, data from in vivo studies were analysed with the non-parametric Mann–Whitney $U$-test (Instat™, GraphPad Software, San Diego, CA, USA). Kaplan–Meier plots were generated using days of animals death (after inoculation of tumour cells) as a criterion, and survival time of animal was analysed for significance by log-rank survival analysis. Significance was defined as a two-sided $P < 0.05$.

### RESULTS

### Anti-tumour effects of PDT combined with G-CSF in C-26 adenocarcinoma and 3LL carcinoma

As it has been reported in previous studies that the presence of neutrophils contributes to the anti-tumour efficacy of PDT (de Vree et al, 1996*a*), we evaluated the extent to which intensive G-CSF therapy is capable of potentiating the anti-tumour efficacy of PDT. G-CSF was administered i.t. for 5 consecutive days, starting 5 days after inoculation of tumour cells. The dose and

© 2000 Cancer Research Campaign

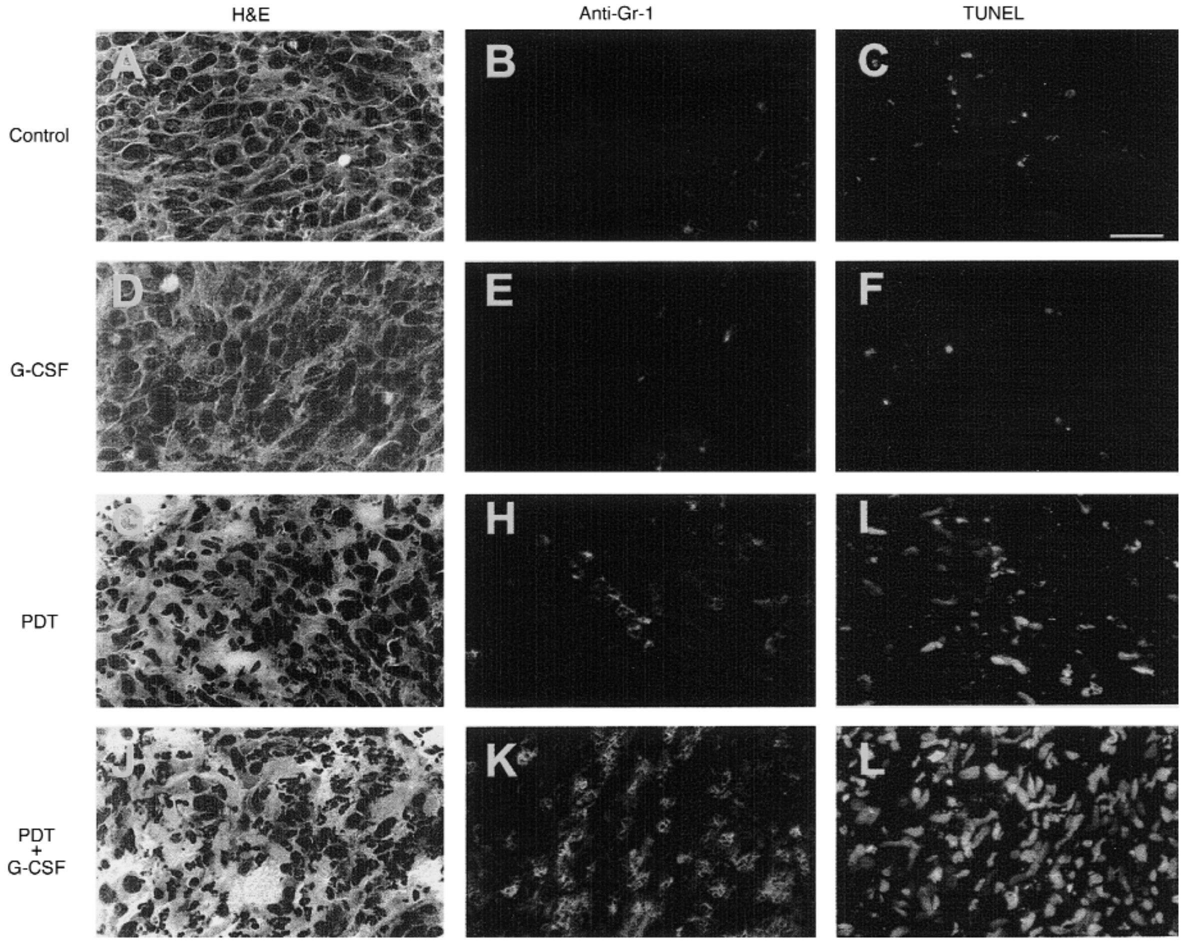

**Figure 2** Histologic analysis, indirect immunofluorescence and TUNEL staining of C-26 tumours. Tumours were obtained from controls (**A–C**), G-CSF (**D–F**), PDT (**G–I**) and PDT + G-CSF (**J–L**) treated mice on day 11 of the experiment (24 h after laser illumination, where applicable). Haematoxylin and eosin staining was performed routinely. On the sections of control (**A**) and G-CSF-treated (**D**) tumours there are densely packed neoplastic cells that form a uniform and solid tumour mass. After PDT (**G**) the tumour architecture is disturbed dramatically, multiple foci of necrosis and/or apoptosis are found, with occasional granulocyte infiltrations. Indirect immunofluorescence (anti-Gr-1) using monoclonal antibodies to detect neutrophils in the tumour specimens reveals single cells in the control (**B**) and G-CSF-treated (**E**) specimens. The number of granulocytes in the PDT-treated tumours (**H**) is evidently greater and confined to focal areas, especially under the epidermis. Importantly, the granulocyte infiltrations are most intensive and distributed throughout the tumour after the combined treatment (**K**). The apoptotic DNA fragmentation was detected by means of terminal deoxynucleotide transferase based, in situ cell death detection kit (TUNEL). There are only single apoptotic cells in tumours from controls (**C**) and G-CSF-treated (**F**) mice. The number of apoptotic cells is greater in tumours obtained from PDT-treated mice (**I**). Tumour specimens obtained after combined treatment (**L**) contain cells that are predominantly either already apoptotic or in the early phases of apoptosis timing of G-CSF administration (1 μg, every 12 h) was chosen based on our previous experiments demonstrating effective stimulation of granulopoiesis in mice (Gołąb et al, 1998*a*). As shown in Figure 1A, 1B, 1C and 1D, PDT produced potent anti-tumour effects against both tumours that were manifested as a reduction of tumour growth and a prolongation of mice survival time. Intensive G-CSF treatment strongly potentiated these effects, further reducing tumour growth. Mice treated with a combined (PDT and G-CSF) regimen survived significantly longer than mice in all other groups. Significantly, 33% of C-26-bearing mice (three of nine) were cured of their tumours (no tumour for 150 days after inoculation of C-26 cells). The cured mice ($n = 3$) rejected rechallenge with even 10 times higher dose ($1 \times 10^6$) of C-26 cells injected into the contralateral footpad on day 150, but did not reject other tumour cells (Ha-*ras*-transformed 3T3 cells), suggesting the development of specific immunity against the treated tumour. Control mice ($n = 6$), injected at the time of rechallenge, developed progressively growing tumours and died within 40 days after inoculation of tumour cells.

On H & E-stained sections, the C-26 tumours from both control and G-CSF-treated mice were composed of solid malignant tissue, with frequent mitotic figures (Figure 2 A and D). The architecture of PDT-treated tumours was dramatically disturbed and neutrophils scattered between residual tumour cells were observed (Figure 2G). However, there were also regions of undisturbed tumour tissue after PDT only (not shown). In contrast, no such regions of undisturbed tumour were observed on sections taken from the tumours after combined treatment, and both tumour

 *© 2000 Cancer Research Campaign*

**Table 1** The influence of G-CSF and/or Photofrin® on haematopoiesis

(A) Changes in peripheral blood leucocyte counts in G-CSF and/or Photofrin®-treated mice

| **Treatment group** | **WBC** $\times 10^{-6}\ \mu l^{-1}$ | **Granulocytes** $\times 10^{-6}\ \mu l^{-1}$ | **Lymphocytes** $\times 10^{-6}\ \mu l^{-1}$ |
|---|---|---|---|
| Control | 6.46 ± 0.52 | 1.16 ± 0.24 | 5.30 ± 0.45 |
| Photofrin | 6.09 ± 0.50 | 0.79 ± 0.13 | 5.30 ± 0.44 |
| G-CSF | 16.59 ± 1.81* | 7.90 ± 1.10* | 8.41 ± 0.92 |
| Photofrin + G-CSF | 20.35 ± 1.87* | 11.38 ± 1.45* | 8.97 ± 0.53* |

(B) The influence of Photofrin® and/or G-CSF on CFU-GM and CFU-GEMM colony formation in the bone marrow and spleens. The numbers values refer to the number of colonies per organ $\times 10^{-3}$

| | **Bone marrow** | | **Spleen** | |
|---|---|---|---|---|
| **Treatment group** | **CFU-GEMM** | **CFU-GM** | **CFU-GEMM** | **CFU-GM** |
| Control | 23.67 ± 1.99 | 78.20 ± 13.47 | 26.00 ± 3.52 | 69.40 ± 5.19 |
| Photofrin | 21.33 ± 2.44 | 109.20 ± 8.39 | 24.83 ± 1.97 | 93.50 ± 7.59* |
| G-CSF | 24.67 ± 1.61 | 168.83 ± 6.06* | 21.67 ± 1.41 | 579.29 ± 17.72* |
| Photofrin + G-CSF | 23.00 ± 3.33 | 205.00 ± 20.67* | 31.67 ± 3.12** | 598.93 ± 48.84* |

Balb/c mice ($n$ = 6–8) were treated with G-CSF and/or Photofrin® in the same treatment schedule as in experiments in which antitumour activity was evaluated. In controls 5% dextrose and 0.1% BSA-PBS were used. Results represent means ± s.e. *$P < 0.05$ versus controls; **$P < 0.05$ versus each of the remaining groups. (Student's $t$-test; two-tailed). The values refer to the number of colonies per organ $\times 10^{-3}$

**Table 2** The influence of G-CSF and/or Photofrin® on peripheral blood neutrophil chemiluminescence

| **Treatment group** | $Cl_{spont}$ **cpm** | $Cl_{stym}$ **cpm** | **Ratio** $Cl_{stym}/Cl_{spont}$ |
|---|---|---|---|
| Control | 3848 ± 459 | 6246 ± 893 | 1.65 ± 0.21 |
| Photofrin | 5186 ± 527 | 25807 ± 3546 | 4.92 ± 0.47* |
| G-CSF | 5919 ± 1190 | 44491 ± 8006 | 9.29 ± 2.36* |
| Photofrin + G-CSF | 9456 ± 1244 | 300716 ± 39344 | 38.27 ± 8.50** |

Balbc mice ($n$ = 8–10) were treated with G-CSF and/or Photofrin® in the same treatment schedule as in experiments in which anti-tumour activity was evaluated. In controls 5% dextrose and 0.1% BSA-PBS were used. The values refer to counts per minute (cpm). The ratio of spontaneous to stimulated chemiluminescence was counted in order to eliminate the differences in neutrophil counts between groups. Results represent means ± s.e. *$P < 0.05$ versus controls; **$P < 0.05$ versus each of the remaining groups (Student's $t$-test; two-tailed)

destruction and neutrophil infiltrations were much more intensive (Figure 2J).

Immunohistochemical staining of tumours taken from mice 24 h after PDT confirmed infiltration of tumours by neutrophils (Figure 2H). Characteristically, the neutrophils were localized mainly in superficial layers of tumours (close to the epidermis). However, numerous neutrophils were also found deeper in the tumour. The neutrophil infiltrations were substantially more intensive in tumours treated with the combined therapy (Figure 2K). These infiltrations were dense throughout the tumour.

In the tumours of mice treated with the combined regimen, multiple sites with apoptotic cells were observed on histological sections. To verify whether the observed cells were truly apoptotic, a TUNEL staining was performed. Indeed, in the sections of tumours treated with PDT, or PDT with G-CSF, numerous apoptotic cells were present (Figure 2 I and L). Importantly, both the neutrophil infiltration and the extent of apoptosis in tumours treated with both PDT and G-CSF together were more intensive than in tumours treated with PDT alone.

### Effects of Photofrin® and G-CSF on myelopoiesis

It has previously been described that Photofrin® at doses used in PDT accelerates myelopoietic recovery in irradiated mice and increases responsiveness of myeloid cells to recombinant myeloid growth factors (Hunt et al, 1995, 1998). Therefore, we decided to examine the effects of co-administration of Photofrin® and G-CSF (at doses used in the experiments with tumours) on the number of peripheral blood leucocytes and colony forming capabilities of bone marrow and spleen cells. Although Photofrin® did not significantly influence the number of peripheral blood leucocytes in our studies it slightly potentiated the effect of G-CSF (Table 1A).

To further investigate the effects of Photofrin® and G-CSF, we examined the influence of these agents, administered either alone or in combination, on the generation of spleen and bone marrow CFU-GM and CFU-GEMM colonies (Table 1B). G-CSF administered at doses used in the treatment of tumours increased the number of bone marrow and spleen CFU-GM colonies. Administration of a single bolus of Photofrin® slightly potentiated

© 2000 Cancer Research Campaign the influence of G-CSF on the number of bone marrow CFU-GM colonies. Moreover, it significantly potentiated the influence of G-CSF on the formation of CFU-GEMM colonies in the spleen.

### Influence of Photofrin® and/or G-CSF administration on luminol-ECL of peripheral blood granulocytes

We also decided to evaluate whether treatment with both agents given alone or in combination affects one of the effector mechanisms of mature granulocytes, namely the production of reactive oxygen species. As expected, G-CSF treatment increased the luminol-ECL of zymosan-activated peripheral blood granulocytes. This effect was further significantly potentiated when mice were given both agents simultaneously (Table 2).

### DISCUSSION

de Vree et al (1996*a*) were the first to suggest that G-CSF could potentiate anti-tumour efficacy of Photofrin®-based PDT. However, in their study, G-CSF was administered at a very low dose and for a short time only. As a result, the retardation of tumour growth was observed on 1 day only in rats treated with Photofrin®-based PDT and G-CSF as compared with PDT-treated animals. Since G-CSF is relatively well tolerated, we decided to administer it at high doses that ensure a strong and consistent stimulation of myelopoiesis (Gołąb et al, 1998*a*).

The results of the present report indicate that treatment with high doses of G-CSF strongly potentiates the anti-tumour effects of PDT. This combined therapy was effective not only in the reduction of tumour growth, but also in the prolonging of mice survival time (Figure 1). Moreover, in C-26 tumour model a combined treatment produced almost 33% cures, an effect not observed in animals of any other treatment groups in our experiments (Figure 1 B and D).

Treatment of tumours with PDT elicits a strong and localized inflammatory response as lymphocytes, granulocytes, macrophages and mast cells are found to rapidly infiltrate PDT-treated tumours (Krosl et al, 1995; Gollnick et al, 1997). A number of cytokines are produced within tumours after PDT. Interleukin (IL)-1β, IL-2 and tumour necrosis factor (TNF) are present in the urine of bladder cancer patients treated with Photofrin®-based PDT (Nseyo et al, 1990). Fragments of PDT-killed tumour cells, inflammatory cytokines and infiltrating leucocytes, capable of engulfing and presenting tumour antigens to T lymphocytes, might create a unique environment for promoting cell-mediated immunity. Administration of G-CSF, by increasing the number and activation status of granulocytes, possibly extends the repertoire of antigen-presenting cells, further facilitating the development of specific anti-tumour immune response (Stoppacciaro et al, 1993). Indeed, it has been reported that activated granulocytes infiltrating tumours are capable of producing IL-1α, IL-1β, IL-8 and TNF, and of cooperating with $CD8^+$ cytotoxic T lymphocytes in the induction of effective anti-tumour immunity (Stoppacciaro et al, 1993; Zilocchi et al, 1998). It has also been previously observed that PDT generates tumour-specific T lymphocytes that can be recovered from distant lymphoid organs, such as lymph nodes or spleen, even after protracted times after light treatment (Korbelik, 1996). Moreover, the effectiveness of PDT in either SCID or nude mice is greatly attenuated as compared with wild-type mice. Adoptive T-cells or bone marrow transfer into nude or SCID mice were effective in delaying the recurrence of PDT-treated tumours (Korbelik et al, 1996).

Although, it cannot be excluded that the observed anti-tumour effects of the combined treatment might result from the G-CSF-induced changes in the level and distribution of Photofrin®, the effectiveness of the combined PDT and G-CSF treatment seems to result from the increased tumour infiltration by neutrophils. G-CSF and Photofrin® are capable of synergistically stimulating the activation of these cells (increased chemiluminescence; Table 2). By inducing increased vascular permeability PDT facilitates tumour infiltration by activated neutrophils that could kill attenuated tumour cells. The striking increase in the number of apoptotic cells in tumours treated with the combination therapy might be a result of increased death of both tumour cells and activated neutrophils that so massively infiltrate the neoplatic tissue (Figure 2L). Apoptotic tumour cells might then be phagocytosed by dendritic cells enabling them to process and present tumour-derived peptides to T-cells, thus inducing the development of anti-tumour immune response (Albert et al, 1998). This response seems to be long-lasting as mice cured of their tumours were able to reject second rechallenge with tumour cells even after 150 days after initiation of the experiment. Moreover, it seems that the immune response is specific, since cured mice were not able to reject another, syngeneic tumour.

Combined PDT and G-CSF treatment could have additional benefits for cancer patients that are also frequently treated with myelosuppressive regimens including radio- and chemotherapy. G-CSF can decrease the duration of neutropenia and the risk of infections in both neutropenic and non-neutropenic patients undergoing chemotherapy (Welte et al, 1996).

Interestingly, Photofrin® alone demonstrates a number of biologic effects. It promotes haematopoietic activity within bone marrow and spleens of normal mice partly through the increased responsiveness to recombinant myeloid and erythroid growth factors (Hunt et al, 1995, 1998). In our studies, we observed increased activation of granulocytes obtained from mice treated with both Photofrin® and G-CSF. These cells revealed an enhanced responsiveness to zymosan, a strong activator of reactive oxygen species production. These effects might be relevant not only in the strengthened anti-tumour activity of granulocytes but also in the enhanced protection of tumour patients against febrile infections.

### ACKNOWLEDGEMENTS

This work was supported by grants D/37 and D/54 from Medical University of Warsaw. Jakub Gołąb and Radoslaw Zagożdżon are recipients of the Foundation for Polish Science Award.

### REFERENCES

Ahmad N, Feyes DK, Agarwal R and Mkhtar H (1998) Photodynamic therapy results in induction of waf1/cip1/p21 leading to cell cycle arrest and apoptosis. *Proc Natl Acad Sci USA* **95**: 6977–6982

Albert MI, Pearce SFA, Francisco LM, Sauter B, Roy P, Silverstein RL and Bhardwaj N (1998) Immature dendritic cells phagocytose apoptotic cells via αvβ5 and CD36, and cross-present antigens to cytotoxic T lymphocytes. *J Exp Med* **188**: 1359–1368

Ben-Hur E, Heldman E, Crane SW and Rosenthal I (1988) Release of clotting factors from photosensitized endothelial cells: a possible trigger for blood vessel occlusion by photodynamic therapy. *FEBS Lett* **236**: 105–108

de Vree WJA, Essers MC, de Bruijn HS, Star WM, Koster JF and Sluiter W (1996*a*) Evidence for an important role of neutrophils in the efficacy of photodynamic therapy in vivo. *Cancer Res* **56**: 2908–2911

 *© 2000 Cancer Research Campaign*

de Vree WJA, Fontijne-Dorsman ANRD, Koster JF and Suiter W (1996*b*) Photodynamic treatment of human endothelial cells promotes the adherence of neutrophils in vitro. *Br J Cancer* **73**: 2908–2911

Dougherty TJ, Gomer CJ, Henderson BW, Jori G, Kessel D, Korbelik M, Moan J and Peng Q (1998) Photodynamic therapy. *J Natl Acad Sci* **90**: 889–905

Faddy C, Reisser D and Marin F (1990) Non-activated neutrophils kill syngeneic colon tumour cells by the release of low molecular weight factor. *Immunobiology* **181**: 1–12

Fingar VH, Wieman TJ, Wiehle SA and Cerrito PB (1992) The role of microvascular damage in photodynamic therapy: the effect of treatment on vessel constriction, permeability, and leukocyte adhesion. *Cancer Res* **52**: 4914–4921

Fingar VH, Wieman TJ and Haydon PS (1997) The effects of thrombocytopenia on vessel stasis and macromolecular leakage after photodynamic therapy using photofrin. *Photochem Photobiol* **66**: 4914–4921

Foster TH, Primavera MC, Marder VJ, Hilf R and Sporn LA (1991) Photosensitized release of von Willebrand factor from cultured human endothelial cells. *Cancer Res* **51**: 3261–3266

Gołąb J, Stokłosa T, Zagożdżon R, Kaca A, Giermasz A, Pojda Z, Machaj E, Dąbrowska A, Feleszko W, Lasek,W, Iwan-Osiecka A and Jakóbisiak M (1998*a*). G-CSF prevents the suppression of bone marrow hematopoiesis induced by IL-12 and augments its antitumour activity in a melanoma model in mice. *Ann Oncol* **9**: 63–69

Gołąb J, Stokłosa T, Zagożdżon R, Kaca A, Kulchitska,LA, Feleszko W, Kawiak J, Hoser G, Głowacka E, Dąbrowska A, Giermasz A, Lasek W and Jakóbisiak M (1998*b*). Granulocyte-macrophage colony-stimulating factor potentiates antitumour activity of interleukin-12 in melanoma model in mice. *Tumour Biol* **19**: 77–87

Granville DJ, Levy JG and Hunt DWC (1997) Photodynamic therapy induces caspase-3 activation in HL-60 cells. *Cell Death Differ* **4**: 623–628

Henderson, BW and Dougherty, TJ (1992) How does photodynamic therapy work? *Photochem Photobiol* **55**: 623–628

Hunt DWC, Sorrenti RA, Renke ME, Waterfield E and Levy JG (1995) Accelerated myelopoietic recovery in irradiated mice treated with photofrin. *Int J Immunopharmacol* **17**: 33–39

Hunt DWC, Jiang H and Levy JG (1998) Photofrin® increasess murine spleen cell transferrin receptor expression and responsiveness to recombinant myeloid and erythroid growth factors. *Immunopharmacology* **37**: 267–278

Kashtan H, Haddad R, Yossiphov Y, Bar-On S and Skornick Y (1996) Photodynamic therapy of colorectal cancer using a new light source: from in vitro studies to a patient treatment. *Dis Colon Rectum* **39**: 379–383

Korbelik M (1996) Induction of tumour immunity by photodynamic therapy. *J Clin Laser Med Surg* **14**: 329–334

Korbelik M and Dougherty GJ (1999) Photodynamic therapy-mediated immune response against mouse tumours. *Cancer Res* **59**: 1941–1946

Korbelik M, Krosl G, Krosl J and Dougherty GJ (1996) Role of host lymphoid populations in the response of mouse emt6 tumour to photodynamic therapy. *Cancer Res* **56**: 5647–5652

Korbelik M, Naraparaju VR and Yamamoto N (1997) Macrophage-directed immunotherapy as adjuvant to photodynamic therapy of cancer. *Br J Cancer* **75**: 202–207

Krosl G, Korbelik M and Dougherty GJ (1995) Induction of immune cell infiltration into murine SSCVII tumour by photofrin-based photodynamic therapy. *Br J Cancer* **71**: 549–555

Krosl G, Korbelik M, Krosl J and Dougherty GJ (1996) Potentiation of photodynamic therapy-elicited antitumour response by localized treatment with granulocyte-macrophage colony-stimulating factor. *Cancer Res* **56**: 3281–3286

Nseyo U, Whalen RK, Duncan MR, Berman B and Lundahl SL (1990) Urinary cytokines following photodynamic therapy for bladder cancer. A preliminary report. *Urology* **36**: 167–171

Ochsner M (1997) Photophysical and photobiological processes in the photodynamic therapy of tumours. *J Photochem Photobiol* **39**: 1–18

Pass HI (1993) Photodynamic therapy in oncology: mechanisms and clinical use. *J Natl Cancer Inst* **85**: 443–456

Reynolds T (1997) Photodynamic therapy expands its horizons. *J Natl Cancer Inst* **89**: 112–114

Stoppacciaro A, Melani C, Parenza M, Mastracchio A, Bassi C, Baroni C, Parmiani G and Colombo MP (1993) Regression of an established tumour genetically modified to release granulocyte colony-stimulating factor requires granulocyte-T cell cooperation and T cell-produced interferon $\gamma$. *J Exp Med* **178**: 151–161

Welte K, Gabrilove J, Bronchud MH and Platzer E (1996) Filgrastim (r-methuG-CSF): the first 10 years. *Blood* **88**: 1907–1929

Zilocchi C, Stoppacciaro A, Chiodoni C, Parenza M, Terrazzini, N. and Colombo, MP (1998) Interferon $\gamma$-independent rejection of interleukin 12-transduced carcinoma cells requires CD4+ T cells and granulocyte/macrophage colony-stimulating factor. *J Exp Med* **188**: 133–143

*© 2000 Cancer Research Campaign*
